# Thermal-Induced Microstructure Deterioration of Egyptian Granodiorite and Associated Physico-Mechanical Responses

**DOI:** 10.3390/ma17061305

**Published:** 2024-03-12

**Authors:** Mohamed Elgharib Gomah, Guichen Li, Ahmed A. Omar, Mahmoud L. Abdel Latif, Changlun Sun, Jiahui Xu

**Affiliations:** 1 Key Laboratory of Deep Coal Resource Mining, School of Mines, China University of Mining and Technology, Ministry of Education of China, Xuzhou 221116, China; mohammedel-ghareeb.12@azhar.edu.eg (M.E.G.); sunchanglun@kict.re.kr (C.S.); tb20020033b3ld@cumt.edu.cn (J.X.); 2 Mining and Petroleum Engineering Department, Faculty of Engineering, Al-Azhar University, Cairo 11884, Egypt; 3 Housing and Building National Research Center, Cairo 11511, Egypt; ahmed.abubakr@hbrc.edu.eg (A.A.O.); mahmoud.lotfy@hbrc.edu.eg (M.L.A.L.)

**Keywords:** Egyptian granodiorite, mineral alterations, microstructure deterioration, physical responses, mechanical responses

## Abstract

Mineral transformations often induce microstructural deteriorations during temperature variations. Hence, it is crucial to understand why and how this microstructure weakens due to mineral alteration with temperature and the correlated physical and mechanical responses. Therefore, in this study, physical, chemical, thermal, petrographic, and mechanical analyses were carried out to comprehend better the thermal behaviors of Egyptian granodiorite exposed to temperatures as high as 800 °C. The experimental results indicate that the examined attributes change in three distinct temperature phases. Strength zone (up to 200 °C): During this phase, the temperature only slightly impacts the granodiorite mass loss and porosity, and the P-wave velocity and E slightly decrease. However, the rock structure was densified, which resulted in a minor increase in strength. After that, the transition zone (200–400 °C) was distinguished by the stability of most studied parameters. For instance, mass and porosity did not significantly alter, and the uniaxial compressive strength steadily increased with an axial failure mode. When the temperature rises, transgranular cracks cause the P-wave velocity and elastic modulus to decrease moderately. The decay zone started after 400 °C and continued to 800 °C. This zone is characterized by complicated factors that worsen the granodiorite properties, lead to color shift, and produce a shear failure mode. The properties of granodiorite became worse because of chemical reactions, structural and crystal water evaporation, rising thermal expansion coefficient variation, and quartz inversion at 575 °C (α to β, according to the differential thermal analysis). Thermal damage greatly affected granodiorite’s physical and mechanical properties and microstructure at 800 °C. As a result, UCS measurements were extremely small with a complex failure pattern, making Vp and E unattainable.

## 1. Introduction

The effects of fire on rocks are relevant to various fields, including geomorphics, built culture, civil engineering, and geotechnical engineering. Fire can cause rapid and long-term damage to rocks and stone structures, making it a significant hazard [1]. Construction materials, for example, can be subjected to temperatures surpassing 700 °C in the event of a building fire [2]. Thermal damage investigations are necessary to link losses in strength and shifts in the appearance of stones due to temperature changes and related mineralogy and texture alterations. Consequently, such information is particularly relevant in fire sensitivity and risk assessment, rehabilitation, and strengthening of rock structures that have been unexpectedly exposed to high temperatures [3,4]. High temperatures can cause substantial changes in rock microstructures [5], the formation of new microcracks [6], and the extension and widening of pre-existing ones [7]. As a result, it can trigger mineralogical, physical, and mechanical modifications in rocks that differ considerably from those observed at room temperature [8]. Therefore, the investigation of temperature impact on the variation in rock’s structural, mineralogical, physical, and mechanical properties focuses on scientists’ ongoing interest in improving fire safety in mines and constructions and restoring burnt monuments and historical buildings [9,10,11,12,13].

Several earlier investigations have focused on evaluating the performance of tunnel rock mass after its exposure to fire. Smith et al. [14] utilized actual fire to record significant catastrophic spalling occurrences in sandstone tunnels at relatively low temperatures. However, they marked a reduction of sandstone uniaxial compression strength (UCS) and elastic modulus (E) when employing an electrical furnace. On the other hand, the investigations on igneous rock tunnels by Nordlund et al. [15] enabled them to determine temperature ranges that significantly changed rock mineralogical, mechanical, and physical characteristics. The findings revealed a strong link between microcrack development, mineralogy, and compression strength reduction. Luo et al. [16] proved that the intrinsic microstructure of rocks was modified, with the range of discrepancy rising with the temperature. Similarly, a substantial series of laboratory experiments on the physical and mechanical characteristics of rocks after exposure to high-temperature conditions have been conducted by various researchers. Huang et al. [17] examined the characteristics of sandstone at temperatures up to 1000 °C. They observed that the most remarkable difference in the P-wave velocities and tensile strength occurred after the high temperature, which is thought to be produced through the absorption by the generated interior cracks. The same outcome was obtained by Sha et al. [18], who explored granite’s wave velocities and microstructure development under open fire.

Furthermore, Gomah et al. [19] studied the preheated longitudinal wave velocity propagating through granodiorite, finding that the wave velocity decreased following heating, and the reduction rate also rose as the temperature increased. In addition, the leading cause of the rocks’ uniaxial compressive strength reduction was the worsening of rock microstructures and the growth of microcracks [20,21]. The volume and porosity of granite [22,23,24], granodiorite [25,26], marble [27], sandstone [28,29], limestone [30,31], and claystone [32] revealed increases with growing temperature, while density and wave velocity dropped. These changes in physical characteristics are fundamentally attached to the deterioration of the rock microstructure. Consistent water escape, dehydration, growing porosity, partial melting, formation, growth, and connection of cracks on grain boundaries, creation and development of micro-cracks, decomposition of minerals, etc., were indications of such modifications in rocks. 

Microscopically, the issue of thermally generated microcracks decreasing granite’s mechanical properties has been investigated. Accordingly, intergranular and intragranular microcracks would generate between and inside particles in the heated rocks [33]. Furthermore, scanning electron microscopy and optical microscopy technologies have been used to disclose the mechanisms of physico-mechanical characteristic deterioration [24,34]. Thermal damage to the rock caused by high temperatures can result in interior cracks beginning and expanding and degrading their mechanical and physical properties [35,36]. Most thermally induced microcracks are usually located at grain boundaries under low temperatures. They are created by bounded changes in temperature gradients within the rock, which cause crack generation and propagation [37]. With growing temperatures, transgranular cracks develop more critically and impact the rock’s overall characteristics [19,38]. Under the impact of high thermal treatment but below the melting temperature of rock minerals, its microstructure reorganizes, new microcracks form, and old ones spread and expand, causing structural damage [39].

Moreover, in crystalline rocks, high-temperature-induced cracks are most visible near quartz borders [40]. The main reason for that is quartz has a higher coefficient of thermal expansion than other minerals [41]. Therefore, to explain and predict variations of rock properties after exposure to the thermal treatment, it is necessary to understand the micro mechanism of the thermal cracking of rocks. In addition, using a combination of thermogravimetry analysis (TGA) and differential thermal analysis (DTA) techniques to examine rock microstructure degradation under temperature impact and the related thermal change in mineral phases and mass loss is very important [42,43,44].

Mineral transformations are the primary source of rock structural and color changes following heating. Furthermore, the degradation of rocks’ mechanical and physical properties after thermal treatment processes is closely linked to their microstructure. Therefore, it is crucial to comprehend why this microstructure weakening results in a loss of strength at high temperatures. Granodiorite is an exceptionally frequent medium- to coarse-grained intrusive igneous rock. Granodiorite is widely distributed and is used in many applications at high temperatures, but it is rarely thoroughly examined under thermal stress in the way that the present work does. Thus, systematic thermal investigation of a new stone is paramount, and this paper tries to fill that gap. The present research aims to study and monitor the impact of high temperatures (200, 400, 600, and 800 °C) on the Egyptian granodiorite rock microstructure and its association with physical and mechanical features based on textural, thermal, and petrographic analyses. Measurements of longitudinal wave velocity, X-ray fluorescence, X-ray diffraction, thermal gravimetric analysis, differential thermal analysis, and optical microscopy are used for this purpose. Moreover, scanning electron microscopy, porosity, and mechanical examination measurements are included in the experimental program for this investigation. High-quality data from this investigation will be used to connect changes in mechanical and physical properties to the microscopic and mineralogical damage mechanisms of granodiorite caused by thermal heating. Simultaneously, the research conclusions will be significant and beneficial in furthering our understanding of thermal degradation in rocks. 

## 2. Materials and Methods

### 2.1. Rock Description

**Figure 1:**
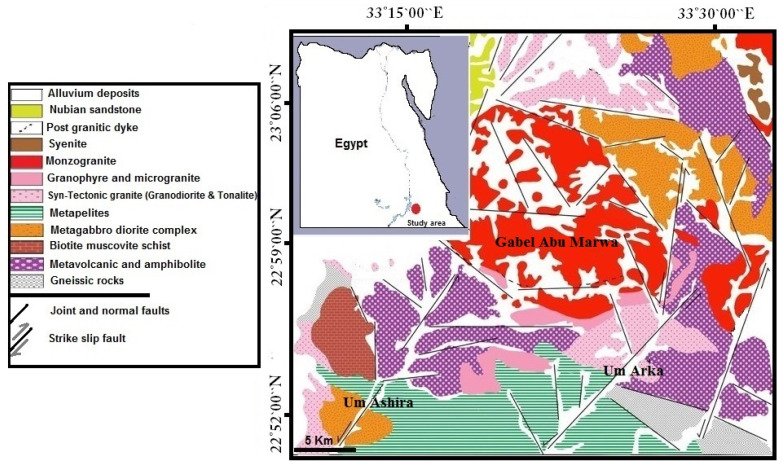
Geological map displaying the studied area of granodiorite samples [48].

The granitoid rock covers extensive swaths of Egypt’s Arabian-Nubian Shield and includes granodiorite and tonalite. It makes up more than 40% of the Eastern Desert’s and Sinai’s underground components [45]. Its composition ranges from quartz diorite and tonalite via granodiorite and quartz monzonite to natural granites and alkaline-peralkaline granites. Several scholars have tried to classify and characterize these granites. Some researchers divided granites into two groups based on their relative age (ancient and younger granites), while others divided granites according to dominant color (grey, red, and pink granites). Likewise, it was separated by localities (Guattarian, Shaitan, and Gharib granites) and apparent orogeny (syn-, late-, and post-orogenic granites) [46,47]. Granodiorite is an intrusive igneous rock like granite in its structure but includes more plagioclase-feldspar than orthoclase-feldspar. The typical granodiorite samples were taken from ancient granite in Egypt’s Eastern Desert (near Gabel Abu Marwa). The studied area is situated at 23°00′ to 23°10′ north latitude and 33°17′ to 33°28′ east longitude (about 130 km Southeast of Aswan). The examined granodiorite rocks surrounding Gabel Abu Marwa (ancient grey granite) were formed by partial melting and absorption of partial melting of metagraywackes at approximately 680 °C [48]. Figure 1 represents the geological map of the sampling locations. Egyptian granodiorite is commonly utilized in modern projects like stairs, hydro-engineering and bridges, road paving materials, construction, and monuments.

When subjected to high temperatures, granodiorite’s mineralogy, microstructure, and associated physical and mechanical aspects will be modified. Thus, the thermal behavior of granodiorite is vital to be investigated. Hence, following the American Society for Testing and Materials (ASTM) D7012–14, granodiorite cylindrical core samples (55.5 mm in diameter and 120 mm long) were obtained. The examined granodiorite was slightly weathered and had a grey color at ambient temperature, with average “P-wave velocities of 5.6 km/s, bulk density of 2610 kg/m^3^, porosity of 0.54%, uniaxial compressive strength (MPa) of 62.7, Young’s modulus of 48.2 GPa, and a Poisson’s ratio of 0.21”. According to the petrographic examination and X-ray diffraction investigations, the major mineral components of Egyptian granodiorite are quartz, plagioclase, K-feldspar, biotite, chlorite, etc.

### 2.2. Experimental Procedures

The fundamental macro- and micro-parameters of prepared specimens, such as uniaxial compression strength, elastic modulus, porosity, wave velocity, mineral composition, and microstructure, at target temperatures, were assessed. Specimens with atypical weights, porosity, and wave velocities were discarded to preserve the test result accuracy and comparability. Then, all samples were separated into five groups of four specimens each, including a reference group that received no heat treatment. After that, the granodiorite specimens were heated to the target temperatures. A high-temperature electric furnace completed the thermal procedure for identified samples (Nabertherm B410). The device’s maximum operational temperature is 1300 °C, and a programmable controller controls the temperature and heating rate. The greater heating rate degrades the rock’s microstructure, modifying its physical, morphological, and mineralogical properties. Consequently, a low heating rate of 5 °C/min has been adopted to avoid any possible thermal shock within the specimens [49]. At four different target temperatures, 200, 400, 600, and 800 °C, thermic effects on granodiorite were studied. To ensure temperature consistency throughout the samples, they were kept in the furnace for 2 h after attaining the appropriate temperature. In addition, the samples were cooled down slowly inside the furnace to eliminate any thermal shock damage during the cooling step. Then, their diameter, height, and mass were re-recorded and compared to their measurements at room temperature. The longitudinal wave velocity is calculated in this study to assess granodiorite thermal damage caused by heating treatment at various temperatures. A pundit PL-2-54 kHz device with two transducers (a transmitter and a receiver) was attached to each end surface of the cylindrical specimen to record P-wave velocity. The time needed for the pulse to pass from the transmitter to the receiver via the specimen’s axial direction was reported. The P-wave was calculated by dividing the specimen length by the pulse travel time through it. The average of three measurements of the P-wave velocity of each specimen was acceptable.

The X-ray diffraction (XRD) analyses were carried out to specify the mineralogical composition and determine the effect of various degrees of heating on the studied granodiorite mineralogy (Manufactured by Panalytical B.V Co., Almelo, The Netherlands). Hence, the studied granodiorite was prepared for analysis as follows: the granodiorite was ground to a <75 μm fraction. It was then examined using the XRD technique using an X′Pert Pro X-ray vertical diffractometer at the Housing and Building National Research Center (HBRC). The investigation was run using graphite monochromatic Cu-kα radiation at 40 kV and 30 mA in 5–50°–2θ. The samples were analyzed using a continuous scanning speed of 2θ/min. The X′Pert high-score software (2006) release of the licensed module, PW3209, was used to process the obtained data and identify minerals. 

Further, SEM was adopted to examine supposable changes in granodiorite microstructure following the thermal treatment (Inspect S. FEI Company, Eindhoven, The Netherlands). In vacuum mode, the FEI Quanta INSPECT-S instrument was used to record the variance in the microstructure with magnifications up to 6000× and an accelerating voltage of 200 V to 30 kV. Furthermore, five samples were examined by the optical light microscope to observe the mineral composition and the internal structure of the granodiorite at room temperature and after exposure to various grades of heating. The granodiorite specimens were impregnated with epoxy resin in a vacuum box (cocking method) and were cut and placed on microscopic glass slides with epoxy. The thin sections were polished using SiC powder (silicon carbide powder) of different sizes (#220, #320, and #600) at the petrography lab in HBRC. After the thin sections were reduced to around 20 μm, they were examined using a transmission polarizing microscope (Olympus BX50, Shinjuku, Tokyo, Japan) equipped with an illuminating source and several magnification scopes ranging from 4× to 40×.

Moreover, the X-ray fluorescence (XRF) technique was utilized at (HBRC) Cairo, Egypt, to establish the chemical composition of the granodiorite. The specimens were ground, and polyvinyl meta-acrylate was used as a binding material. The samples were pressed manually up to five tons on a Herzog packing machine (type TP 60/2D, Osnabrück, Germany). In the present work, quantitative determination of the major oxides was accomplished by computerized X-ray fluorescence (Phillips PW 1400 Spectrometer, Eindhoven, The Netherlands).

Recently, thermocouples, platinum resistance thermometers, and thermistors have been commonly used in thermal analysis tools. The sample may absorb (endothermic) or release (exothermic) heat as it transforms. Therefore, as confirmation tools on the samples under examination, thermogravimetry analysis (TGA) and differential thermal analysis (DTA) were utilized to assess the mass loss and phase changes of granodiorite throughout the heat treatment. For the thermal evaluations, a computerized DT-50 thermal analyzer with a potentiometric recorder (123 T) was utilized (Shimadzu Co., Kyoto, Japan). The heating temperature was 1000 °C with a 20 °C/min rate for DTA and TGA under a nitrogen atmosphere (30 mL/min). Additionally, the granodiorite specimens were subjected to uniaxial compression testing. The sample is exposed to axial stress at a displacement control rate of 0.05 mm per minute, with three specimens tested at each temperature.

## 3. Results

### 3.1. Physical Analyses

Physical parameters such as porosity, mass loss, attenuation of ultrasonic waves, and the appearance measured following thermal treatments are beneficial indicators of the degree of deterioration generated in the rock specimens [50]. As a result, all samples’ original properties (weight, volume, P-wave, hue, etc.) were measured before heating to establish a baseline for comparing the variance caused by thermal treatments. Each specimen’s mass, porosity, and P-wave velocity were assessed individually and then as an average across all samples undergoing the same temperatures.

#### 3.1.1. Connection between Porosity and Mass Loss

**Figure 2:**
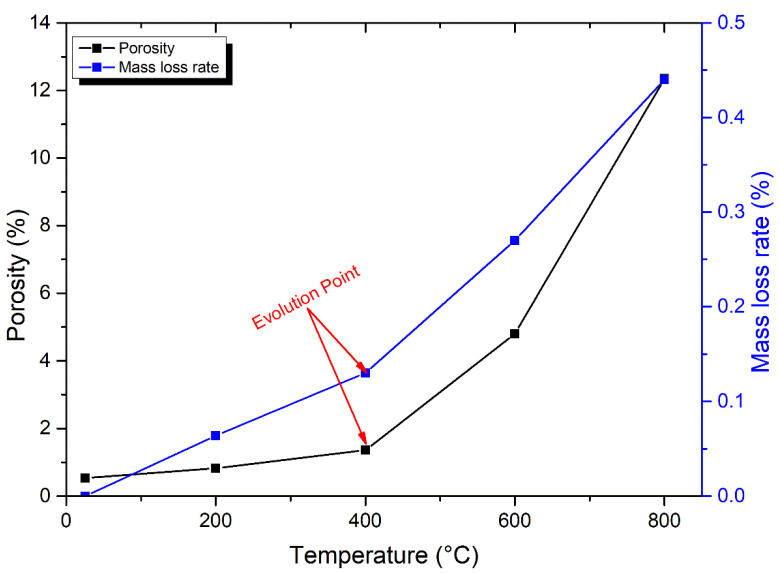
The relation between porosity and mass loss ratio with temperature for granodiorite samples following different thermal treatments.

The evaporation of various forms of water is mostly responsible for the weight loss of rocks after thermal treatments. For example, 100 °C, 300 °C, and 500 °C are the evaporating temperature limits of crystal, structural, or zeolite water, respectively [51]. Further, one of the most significant physical variables affecting the mechanical properties of rocks and the density of cracks is porosity. Therefore, temperature-related changes in mass and porosity were observed in Figure 2. Generally, the mass of the samples almost stays constant up to 400 °C (with a minor rise at T = 400 °C), after which it drops dramatically. For example, the mass-loss rate changed from 0.06% at 200 °C to 0.13% at 400 °C. On the other hand, between 25 and 400 °C, there was a modest increase in porosity. For instance, the porosity extends from 0.54% at room temperature to 0.83% at 200 °C. Then, at 400 °C, a moderate rise of 1.36% was observed. That indicated that the various forms of water that escaped from granodiorite throughout this stage were very limited, such as structural, crystal, and zeolite water.

Following 400 °C, an expansion in granodiorite pore volume and a drop in mass occurred due to the deconsolidation between particles and water evaporation at high temperatures. Hence, there was an apparent increase in mass losses and rising porosity, which indicated a substantial increase in thermally generated cracks. A reduction of 0.27% and 0.44% in mass and a rise of 4.8% and 12.35% in porosity were indicated at 600 °C and 800 °C, respectively. Hence, as shown in Figure 2, a good link between granodiorite mass loss and porosity at the various heat treatment temperatures reveals the evolution point is 400 °C.

#### 3.1.2. Connection between Porosity and P-Wave Velocity 

**Figure 3:**
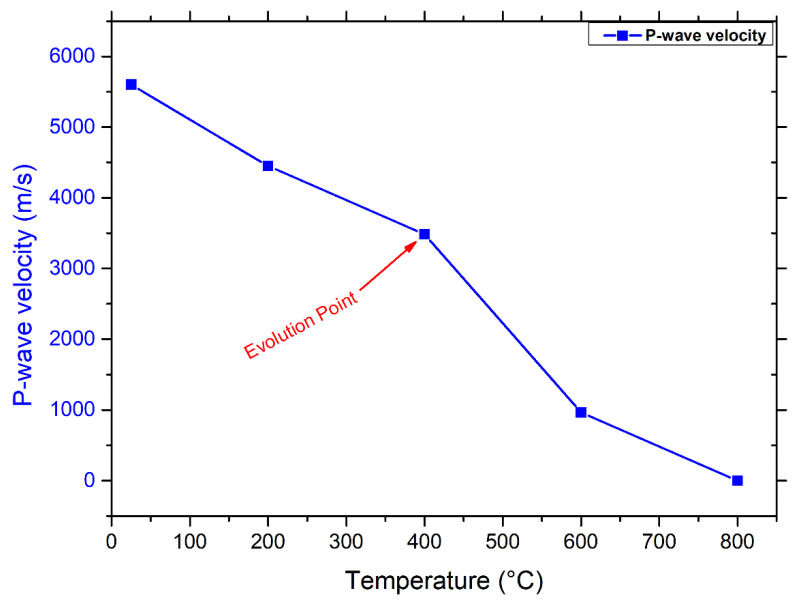
Relationship between temperature and P-wave velocity for granodiorite samples after various thermal treatments.

Since it is closely linked to the physical characteristics of rock, such as density, water content, structural properties, and porosity, ultrasonic wave velocity variations through the rock material can provide information on the rock’s quality and durability, including assessments of porosity, mechanical properties, cracks, and deformation behavior [52]. Hence, the degradation rate of rocks subjected to thermal processes may be accurately measured using ultrasonic pulse velocity [53]. Figure 3 shows that velocity and temperature have a negative relationship. 

There was a moderate (39%) decline in velocity between 25 and 400 °C. Temperature induces rock cracking by thermal stress and increases interior pores generated by water loss. P-wave velocity reduces with increasing temperature, and this decline becomes more pronounced. Consequently, after a high-temperature treatment at 400 °C (the evolution point of Pv), the acceleration decreased drastically (39–85%) around 3.4–0.96 km/s for specimens at 600 °C This suggests that several new thermally induced cracks had formed during the heat treatments above 400 °C, and the granodiorite samples had suffered significant damage. As is known, for quartz-dominated rocks, the most considerable and drastic shift in porosity occurs after 600 °C, at which the cracking exhibited inside and outside of the quartz grains results from the transition of quartz at 573 °C. Hence, the porosity of the sample increased quickly at 600 °C, by nine times the values of the samples at room temperature. Thus, measuring waves propagating velocities at 800 °C was problematic due to the severity and deepness of thermal cracks after 600 °C (assumed 0 m/s). Besides, as expected, the ultrasonic velocity was inversely proportional to the porosity, which increased as wave propagation speed decreased. These observed increases in porosity and degradation inside the material due to thermal cracking are consistent with delays in the P-wave velocity first arrival time for each temperature level. 

#### 3.1.3. Appearance

**Figure 4:**
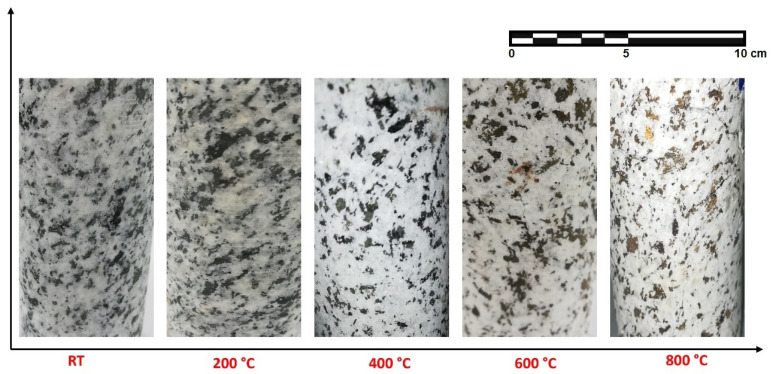
Granodiorite appearance at various temperatures.

Following thermal treatments, color and surface crack initiation are commonly observable qualities used to assess rock. Engineers can use the stone’s color after it has been exposed to temperatures to evaluate the internal damage due to the temperature. At room temperature, the hue of the granodiorite samples was grey when viewed with the naked eye, as shown in Figure 4. According to observations, the color of the oven-dried specimen appears to be grey and kept the same, and no surface cracks were discovered up to 400 °C. Gomah et al. [19] noted that higher temperatures cause granodiorite materials to visually change in color and exhibit some external features like microcracks and volume expansion. Hence, the color lightened and paled to a shiny grey with a reddish tint after 400 °C (Figure 4). 

The color shift at high temperatures may be due to chemical reactions in the constituent minerals caused by temperature [4]. For example, the principal reason for the pale color exhibited at temperatures over 500 °C is the creation of new phases, which is corroborated by the loss of hydroxyl, found in hydrated minerals that did not entirely lose water throughout drying. Since biotite is a dark-colored mineral made up of Fe^2+^ and other elements, it turned brown after 400 °C. Hence, during high-temperature catalytic action, oxygen oxidizes Fe^2+^ to Fe^3+^, and the hue of the biotite is altered. That negatively affected the adhesion force between the granodiorite-forming minerals. Further, between 600 and 800 °C, dehydration of iron oxides provides the specimens with a little golden tinge. Also, the volumetric expansions peaked in that range, and the quartz transition from an α-quartz to β- quartz phase at around 575 °C, as confirmed by DTA later. Hence, at 600 °C, cracks appeared on the surfaces of granodiorite due to substantial thermal damage. Furthermore, cracks increased in size as the temperature rose to 800 °C, with significantly greater length and depth than cracks formed at 600 °C.

### 3.2. Mineralogical Analyses

**Figure 5:**
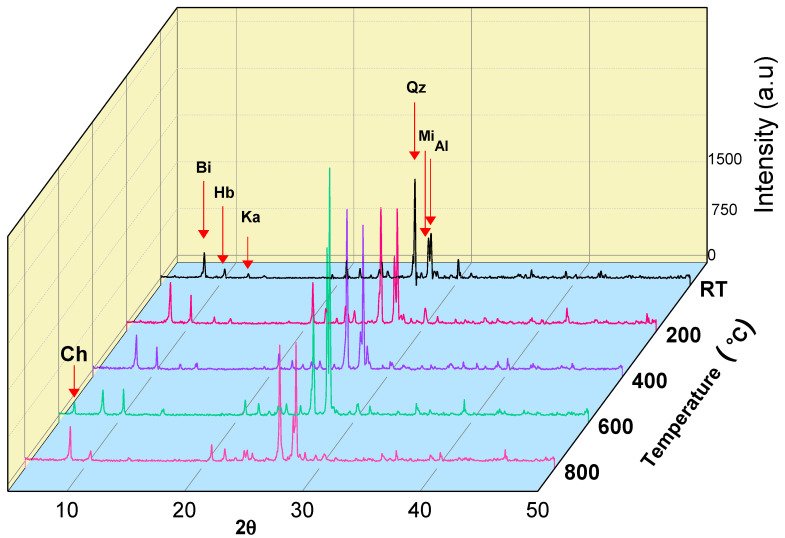
XRD analysis of granodiorite with different heating temperatures from 25 to 800 °C.

The technique of X-ray diffraction (XRD) is widely managed in structural engineering to examine the mineralogical behavior of rocks. Hence, it was applied to investigate how the mineral content of rock materials varies during thermal treatment [54]. Firstly, the studied granodiorite was examined by an X-ray diffraction tool at room temperature. Then, the temperature increased progressively up to 800 °C. The obtained results from the X-ray diffraction pattern (Figure 5) revealed that the mineralogical composition of the studied granodiorite at room temperature is Biotite (Bi), Kaolinite (Ka), and Hornblende (Hb), which represents the amphibole group, Quartz (Q), Microcline (Mi) “potash feldspar”, and Albite (Ab), which represent Plagioclase. At 200 °C and 400 °C, XRD spectra show the same mineralogy. However, the main biotite peak (corresponding to the (001) plane) moves towards higher angles, which reflects a decrease in the network spacing (contraction) [55]. In addition, biotite plays an important role in accommodating the stresses caused by the mineral thermal expansion mismatch [56], which explains why granodiorite withstands the thermal effect and the absence of surface cracks in the specimens. 

However, some internal reactions may have occurred after that temperature range, which could be the primary cause of granodiorite microstructure degradation. For example, at 600 °C, XRD spectra show the disappearance of kaolin peaks, where it transformed into metakaolin. Furthermore, quartz crystals converted from alpha to beta quartz crystals, accompanied by an increase in crystal size [57]. In addition, peaks of chlorite appear at 600 °C, which indicates transforming parts of biotite crystals into chlorite crystals. Thus, because biotite is transformed into chlorite and quartz volume changes, granodiorite loses its ability to absorb the internal stresses, so micro surface cracks appear in the hand specimen. At 800 °C, XRD spectra show the presence of quartz, biotite, albite, microcline, and hornblende and the disappearance of chlorite peaks. Quartz crystals, after transitioning from the alpha to beta phase (>600 °C), start to contract (quartz volume recovery), which explains the creation of intergranular cracks, which produce a loss of integrity of the rocks [55]. 

In addition, as seen in Table 1, the XRF analyses of granodiorite detected a proportionate change in oxides as a function of temperature, mainly notable at 400 °C, representing a threshold point. SiO_2_ was the most prevalent element at all temperatures, followed (by far) by Al_2_O_3_. Further, Fe_2_O_3_, CaO, Na_2_O, MgO, and K_2_O also have significant weights. The minerals in granodiorite (magnetite, biotite, and feldspars) experience chemical reactions between 500 and 600 °C, distinguished by volume increases, bearing capacity decreases, connectedness increases, and wave velocity mutations [58]. Hence, more oxygen can be absorbed into the rock when the temperature rises above 400 °C due to the thermal reactions [59], as shown in Table 1. As a result, the granodiorite’s microstructure weakened after 400 °C.

**Figure 6:**
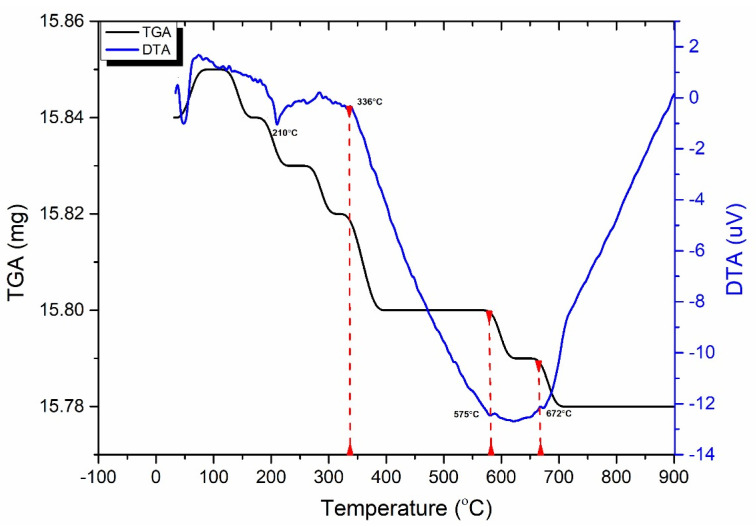
Thermal gravimetric analysis (TGA) regarding the mass loss ratio and differential thermal analysis (DTA) for granodiorite.

Thermal analysis is a critical indicator for identifying any thermal changes that occur due to chemical or structural transformations during the thermal heating of rocks. Consequently, such research is beneficial in determining phase change and recrystallization of mineralogical composition. Thermogravimetry analysis (TGA) evaluates mass loss as a function of temperature [43]. In contrast, differential thermal analysis (DTA) compares the quantity of heat needed (in microvolts) to raise the temperature of a specimen to that of the reference material [44]. Hence, TGA and DTA were used to determine mass loss and phase changes of granodiorite, respectively, throughout the heat treatment. The observations in Figure 6 reveal that temperature has only a minor impact on granodiorite mass. This is due to the prevalence of quartz in granodiorite and the small amount of water present.

Nevertheless, Figure 5 depicts that the mass-loss rate alteration can be split into three temperature ranges. The interior structure of granodiorite absorbs water vapor trapped in confined pores that cannot escape at temperatures between 25 and 336 °C, causing the exothermic effect. That results in a modest increase in mass [60], represented by the slight shift in the TGA curve between 25 to 70 °C. Between 70 and 130 °C, the TGA curve reveals a constant trend with temperature.

Then, a moderate decline in the TGA curve beyond 130 °C was recorded, indicating high mass-loss rates up to 336 °C. After this temperature point, the second phase of transition began, with the quartz transformation occurring at around 575 °C. Then, in the third stage of granodiorite, other mass reduction occurred up to 700 °C, and the max weight of the powdered granodiorite specimen decreased by 0.38% at 700 °C. However, no more mass loss is evident between 700 °C and 900 °C, and the TGA curve becomes constant. Additionally, the DTA curve presents insight into phase transitions when minerals are exposed to high temperatures. Figure 6 shows the differential thermal analysis (DTA) results on granodiorite specimens. Up to 336 °C, the disintegration and evaporation of the structural water cause a drop in the DTA curve, and the oscillation in the curve indicates that small amounts of clay minerals were present. Hence, the accompanying interactions do not cause a notable change in the curve’s form. In contrast, after this temperature phase, a tremendous decline in the DTA curve was discovered, and the findings show that granodiorite underwent a low (α) to high (β) quartz phase transformation at around 575 °C. Furthermore, the following peak, about 672 °C, represents the (partially) melting mark in samples via chemical reactions in minerals such as kaolinite and magnetite [61].

### 3.3. Petrographic Analyses

**Figure 7:**
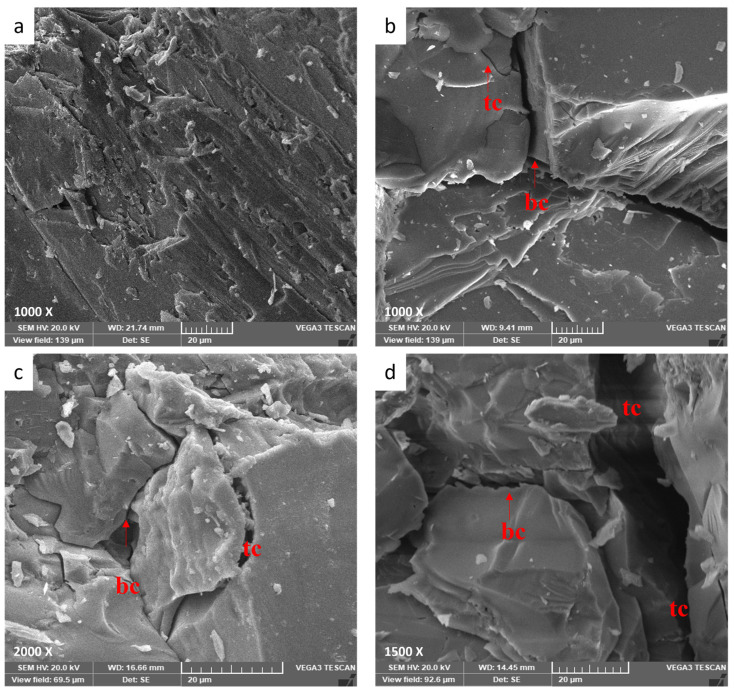
SEM images of granodiorite at room temperature (**a**) and various thermal treatments, 400 °C (**b**), 600 °C (**c**), and 800 °C (**d**). Note that (bc) is boundary cracks and (tc) is transgranular.

The scanning electron microscopy (SEM) technology offers a visible way to study the microstructure developments of rock by scanning the sample with an electron beam. SEM pictures may relate to immediately acquiring grain size, crack developments, holes, and mineral morphology [62]. As a result, it is widely utilized to assess the thermal crack extension, the quantifiable micro-crack densities, and the enlargement caused by heat treatment [49]. As illustrated in Figure 7a, there are nearly no microcracks within the granodiorite specimen when there is no thermal effect and the grains are securely cemented together. 

Once granodiorite is heated, SEM can detect micro-defects of increasing magnitude. For instance, heat expansion produces grain splitting at grain boundaries when the imposed temperatures rise to 400 °C, and a considerable number of microcracks, particularly intergranular microcracks, can be observed, in addition to the sight of some trans-granular microcracks (Figure 7b). When the temperatures were raised to 600 °C, trans-granular microcracks and intergranular cracks were seen in the specimen, and fissure expansion was easier to see (Figure 7c). Furthermore, the texture of granodiorite has deteriorated. Between 600 °C and 800 °C, high temperatures cause inhomogeneous thermal expansion of the mineral particles or phase transition of some of the mineralogical components in granodiorite, resulting in internal stress and microcracks. Intergranular and intragranular microcracks grew in length and width, resulting in granular disintegration. Accordingly, substantial damage to the granodiorite structure and fissures became increasingly broad and interconnected (Figure 7d). 

**Figure 8:**
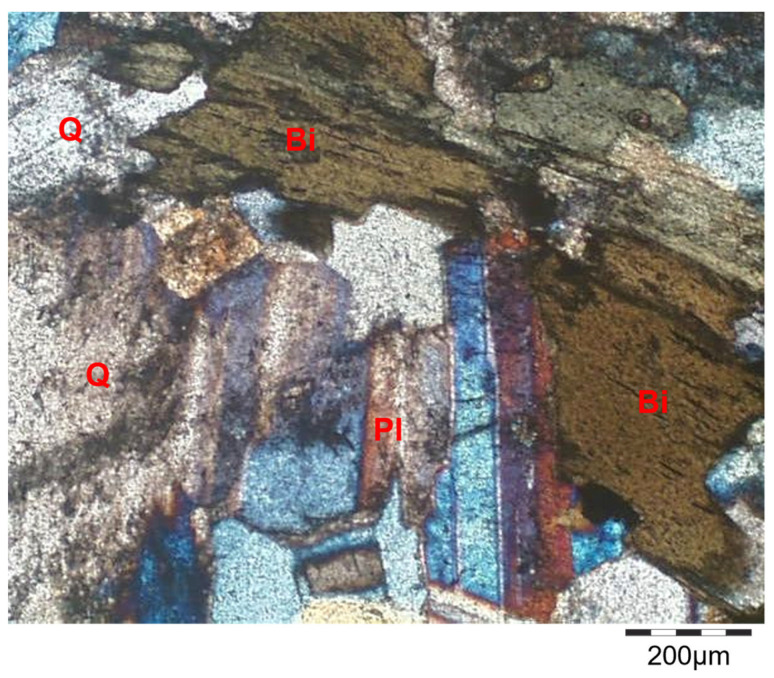
Photomicrograph of an equigranular texture of granodiorite at room temperature. Abbreviations: Q—quartz, Pl—plagioclase, and Bi—biotite.

**Figure 9:**
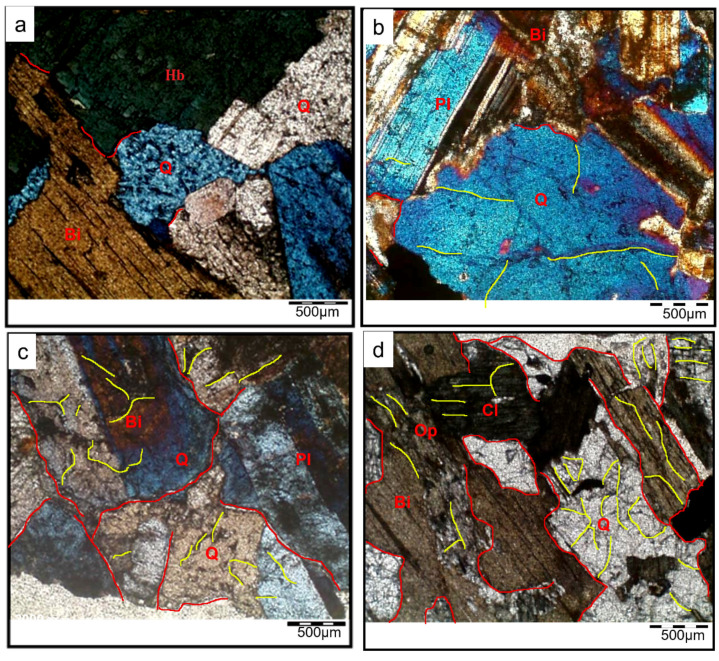
Photomicrographs show different thermal changes in the microstructure of the granodiorite at different temperatures: 200 °C (**a**), 400 °C (**b**), 600 °C (**c**), and 800 °C (**d**). Abbreviations: Q—quartz, Pl—plagioclase, Hb—hornblende, Bi—biotite, Op—opaque minerals, and Cl—chlorite.

In addition to SEM, the mechanisms of structural deterioration were explored using optical microscopic observations on thin sections of granodiorite specimens at room temperature (Figure 8) and after the various heat treatments (200, 400, 600, and 800 °C) shown in Figure 9. Petrographically, at room temperature (RT), the studied granodiorite samples essentially consisted of medium to coarse grains of quartz, plagioclase (albite), Equigranular hornblende, and biotite (Figure 8). Further, the Egyptian granodiorite was slightly weathered during the microscopic examination, as shown in Figure 8. The fabric was well arranged when the specimen was not exposed to thermal treatments; however, it was restricted to some quartz crystals appearing as traces or be (faint), and no microcracks were visible. Quartz appears as an anhedral, cloudy, medium- to coarse-grained crystal with a wavy extinction and grey interference color that occupies most of the intercrystalline spaces among the studied granodiorite constituents. Most plagioclase (albite) occurs as subhedral crystals; they exhibit lamellar twinning and oscillatory zoning; some plagioclase crystals are slightly altered to sericite. On the other hand, biotite presents as flaky and irregular subhedral brown prismatic crystals with a strong pleochroism and parallel extinction (one set of cleavage). 

Likewise, petrographic investigation plays an important role in monitoring the thermal changes in the main microstructure of the granodiorite under certain different degrees of heating (200 °C, 400 °C, 600 °C, and 800 °C), respectively. Alterations in rock microstructures, such as the formation and development of micro-fissures, may result from heat treatments. The optical microscopic observations exhibited that at 200 °C, most of the granodiorite microstructure remains intact. However, due to unequal thermal expansion of minerals, rare micro-cracks start to appear around some boundaries of crystals (boundary cracks) symbolized (‘bc—red lines’) as in (Figure 9a). Variations in thermal expansion coefficients generate stresses, which create microcracking and a deterioration in the material’s characteristics. Thus, with the continuous temperature increase to 400 °C, the uneven thermal expansion between minerals increased, and many intergranular cracks were discovered after thermal treatment (Figure 9b). Furthermore, transgranular cracks (‘tc—yellow lines’) begin forming, especially within the quartz particles.

The number of thermal microcracks in the granodiorite specimens went up as the temperature went up, and they spread widely and merged inside the samples. Hence, at 600 °C, a cracking network (boundary and intragranular cracks) was observed within most of the granodiorite components. At this temperature range, the degree of alteration for different minerals increased. Plagioclase and biotite formed more clays, chlorite, and sericite. 

**Figure 10:**
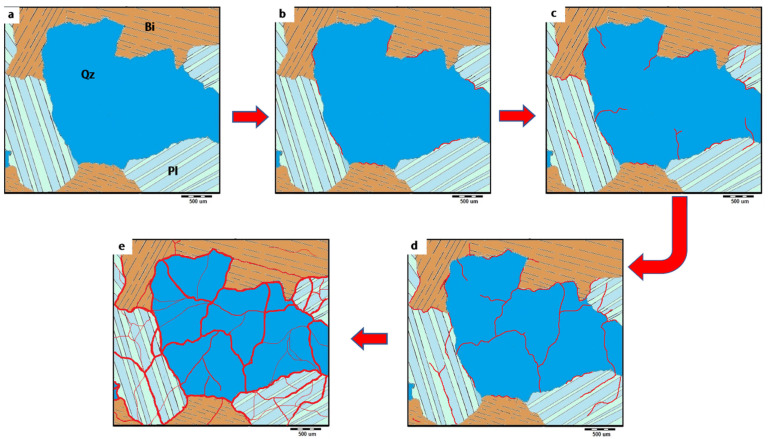
A simulation model of cracks evolution in granodiorite under the different temperatures, (**a**) RT, 200 °C (**b**), 400 °C (**c**), 600 °C (**d**) and 800 °C (**e**).

When the samples were heated to 800 °C, a dense network of microcracks eventually formed, and the number and width of the microcracks all increased (Figure 9d). Furthermore, a simulation model (Figure 10) has been proposed to simplify the evolution of thermal-induced microcracks with increasing temperature. According to the granodiorite petrographic study, micro-cracks at grain boundaries and transgranular micro-cracks made the crack rate faster as the temperature rose. Microcracks develop through weak planes in feldspar, like cleavages and twin planes. Plagioclases also frequently have secondary mineral phases in their nuclei. Furthermore, the large single grain of quartz is also fractured into fewer sub-grains, leading to quartz polycrystallization, mainly caused by severe thermal stress generated by excess heating (Figure 9d).

### 3.4. Mechanical Analyses

#### 3.4.1. Uniaxial Compressive Strength

**Figure 11:**
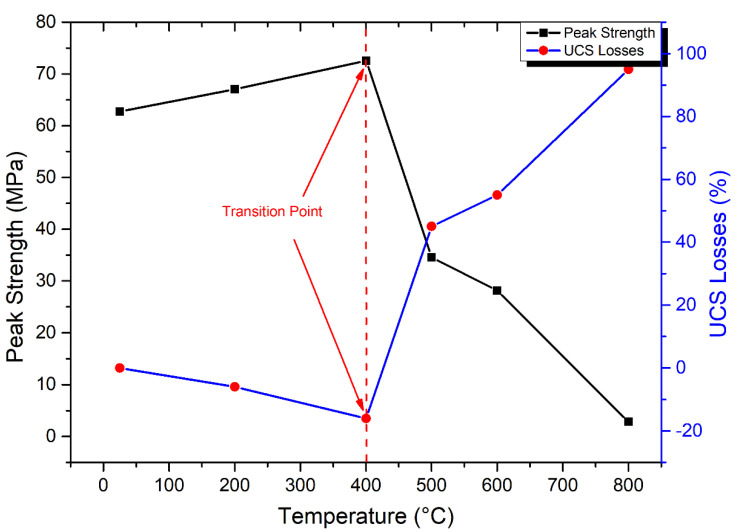
Impact of thermal treatments on the compressive strength of granodiorite.

From room temperature to 800 °C, the compressive strength of granodiorite was studied (Figure 11). Under a certain temperature range, heat treatment can improve rock strength by generating plastic expansions of minerals and enhancing friction between mineral grains [3]. Thus, the granodiorite showed a gently rising trend even at 400 °C. For instance, maximum stress increased by 4 MPa at 200 °C and 10 MPa at 400 °C, respectively, to the room temperature value of 63 MPa. Once the temperature reached 400 °C, the associated effects of thermal stress and applied compressive stress resulted in a considerable increase in the formation of new micro-fractures, ultimately leading to the onset of deterioration of the granodiorite sample. The transition point of granodiorite compressive strength decay started at 400 °C, and UCS fell dramatically from 73 MPa to 28 MPa at temperatures between 400 °C and 600 °C, a loss of 55%. At 800 °C, the peak stress dropped to 2.8 MPa, indicating that numerous minerals began to dissolve and produce new microcracks, causing substantial macro-structural deformation in the granodiorite.

#### 3.4.2. Elastic Modulus

**Figure 12:**
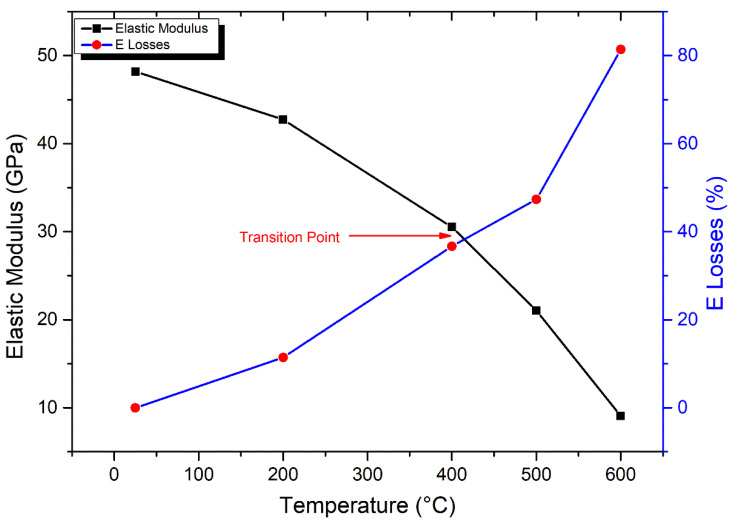
Impact of thermal treatments on the elastic modulus of granodiorite.

As shown in Figure 12, the curves of elastic modulus display a gradual decrease with increasing temperatures. The elastic modulus (E) of granodiorite rocks peaked (48.2 GPa) at room temperature, according to empirical data shown in Figure 12. From 25–200 °C, there is a small decrease in E, with values dropping by 11% due to the evaporating of inherent water from microcracks and pores by thermal treatment. A transition point of E measurement was detected at 400 °C, at which a moderate drop in E value of 37% was recorded. Between 400–600 °C, such a temperature region is dominated by the initiation and expansion of intergranular and transgranular cracks, which increases crack density and porosity. Hence, there was a rapid drop in the elasticity of samples, and E recorded a drastic reduction of 81% at 600 °C. Granodiorite specimens were severely degraded at 600 °C, as demonstrated by the lower values of their UCS at this range of temperature. E readings were therefore unfeasible at 800 °C.

#### 3.4.3. Thermal Crack Mechanism of Granodiorite 

**Figure 13:**
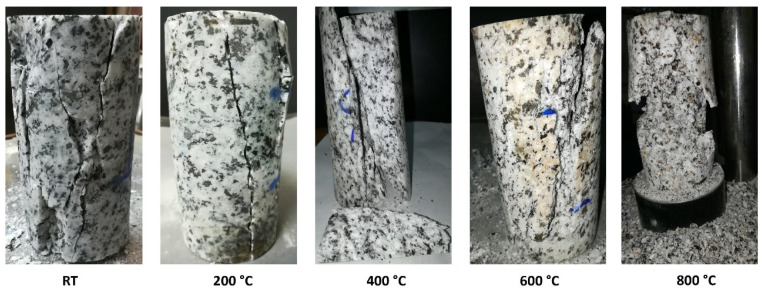
Failure pattern of granodiorite in uniaxial compression tests following different temperature treatments.

Due to being made up of several mineral crystals with different physical qualities, such as being non-continuous, heterogeneous, and so on, the granodiorite grain structure of the rock will alter after being exposed to various high temperatures, resulting in diverse failure modes. Higher temperatures worsen rock qualities and cause more local microcracks, which grow and merge into macrocracks during compression loads. Figure 13 depicts the various rock failure scenarios observed during uniaxial compression experiments. Rock failure mode changes from simple splitting at 25 and 200 °C to multi-splitting surfaces parallel to axial force at 400 °C, predominantly generated by tensile cracks. When comparing the failure modes at room temperature and 400 °C, the failure mode of granodiorite at T = 400 °C is quite comparable to that at T = 25 °C. In contrast, the crack thicknesses of granodiorite at T = 400 °C are higher in several local places. The growth and coalescence of tensile cracks inside thermally treated specimens and their propagation in the stress direction lead to motivational axial splitting [44]. 

As shown in Figure 13, once the granodiorite specimen has thermally generated cracks, these fissures can directly affect the fracturing development process, which is different from samples at lower temperatures. The granodiorite sample experiences a phase transformation at 600 °C; when the temperature hits 575 °C, the cohesiveness between the particles weakens, and the shear fracture propagates and accumulates. As a result, the material has more cracks (Figure 9c,d) and exhibits the optimal shear failure mode at 600 °C. Hence, the evolution transition temperature for granodiorite was 400 °C. Further, because the weakening places created thermal cracks that have broken their cohesiveness at higher temperatures, a relatively low shear stress concentration at grain bonds is enough to break these weakening positions, resulting in frictional slides and grain rotations. The rock undergoes complex transformations as the temperature hits 800 °C, including evaporating the constituent water and the phase transition of quartz and mineral element breakdown. Hence, thermal damage led to the enormous fracture process of granodiorite at 800 °C.

### 3.5. Thermal Damage Factor

This study examined granodiorite’s physical and mechanical properties concerning microstructural alteration after exposure to high temperatures. In geotechnical engineering, damage mechanics has recently been used as an innovative method for understanding rock thermodynamics. Different criteria can be used to forecast the intensity of the thermal damage characteristic of rock. Because *Vp* and *E* are parameters that have excellent correlations with temperature, these values were employed as a thermal damage factor to forecast the thermal effect of granodiorite in this study, as revealed in Equations (1) and (2):

(1) $$D\left(T\right)E=1-\frac{E_{T}}{E_{o}}$$

(2) $${{D\left(T\right)Vp=1-(Vp}_{T}/{Vp}_{o})}^{2}$$

**Figure 14:**
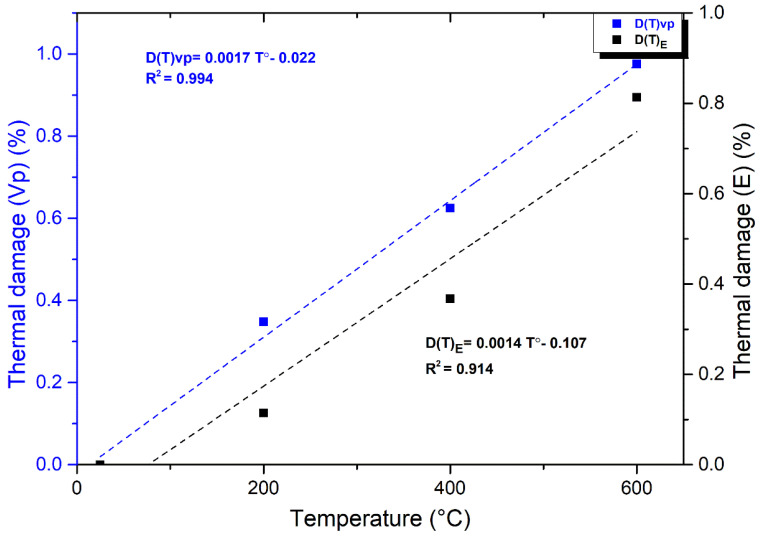
Thermal damage of granodiorite expressed through P-wave velocity and Elastic modulus.

The rock thermal damage variances reflected changes in the rock microstructure after thermal treatment. Both thermal damage *D*(*T*)*Vp* and *D*(*T*)*E* of granodiorite grew with the final heating temperature and adopted a similar pattern. Nonetheless, because the P-wave velocity was more sensitive to temperature than the elastic modulus [63], as shown in Figure 14, the curve of thermal damage *D*(*T*)*Vp* was stronger than *D*(*T*)*E*, with a correlation coefficient greater than 0.99, characterizing this relationship.

Furthermore, due to significant heat damage within the granodiorite microstructure generated by the elevated temperature, the examined variables’ thermal damage factor peaked at 600 °C. Consequently, that interprets the impossible measurements of *Vp* and *E* at 800 °C because of the significant thermal damage in the granodiorite samples after 600 °C.

## 4. Discussion

**Figure 15:**
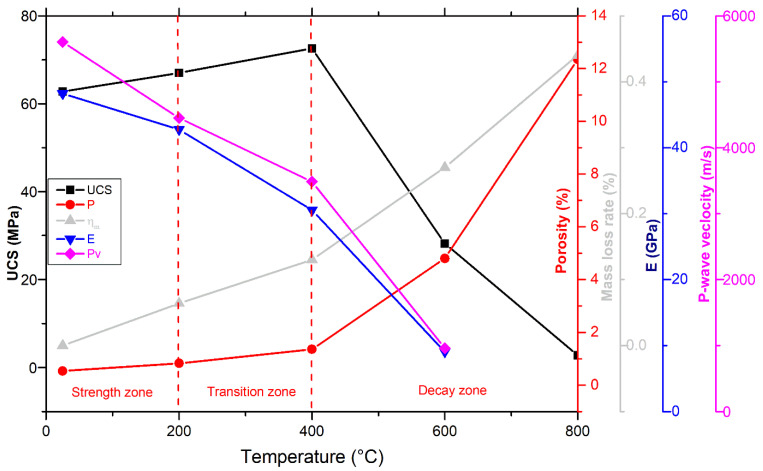
Correlations between physical and mechanical parameters at various thermal treatments.

In high-temperature thermal applications, it’s necessary to evaluate the influence of high temperatures on rock mineralogy and their microstructure and physical and mechanical properties response. According to previous investigations, thermal treatment promotes mineral expansion and chemical reactions, which leads to the beginning and spread of microcracks and microfracture. Thermal treatment leads grains to expand, with different minerals exhibiting various thermal expansion properties, resulting in microcracks between or inside the grains. Furthermore, the water within granodiorite changes forms under multiple temperatures, such as crystal, structural, or zeolite water, which would escape at different temperatures. Consequently, increasing the number of micropores may damage the mineral silicate framework [58] and influence the microstructure of granodiorite. Thus, this research focused on studying granodiorite’s physical and mechanical properties related to mineral composition alteration and microstructure deterioration due to various thermal treatments. Besides, changes in porosity, P-wave, mass, and mechanical characteristics such as UCS and E can be correlated and compared to ambient temperature. Based on the results, the behavior of thermally treated Egyptian granodiorite may be divided into three stages, as shown in Figure 15.

Between 25 and 200 °C (Strength zone): initially, under natural circumstances, the microstructures of the specimens showed good durability. Accordingly, all physical and mechanical parameters provided the highest values. The granodiorite has minimal structural changes for this temperature range owing to a small number of intergranular cracks between mineral particles at 200 °C (Figure 9a). Therefore, there was a slight increase in porosity and a small decline in P-wave velocity and E. Furthermore, as the water content of granodiorite is limited, as confirmed by TGA analysis (Figure 6), the oscillations in the rate of granodiorite mass loss with temperature were minor. Temperature can cause cracks to seal, reducing the number of microcracks and increasing densification, resulting in increased strength [39]. Hence, the thermal treatment firstly increased the compressive strength of the granodiorite by sealing the original cracks created by uneven mineral expansion and densifying the rock structure during heating, resulting in stronger adhesion between the grains. So, moisture evaporation decreases grain sliding at this stage, increasing friction, and then the recently formed friction prevents slight deformation or motion between the grains [3].

From 200 to 400 °C (Transition zone): there was no visible increase in microcracks after heat treatments (Figure 7b and Figure 9b) and, hence, no significant change in porosity or mass. Also, the TGA curve began to fall gradually, showing moderate mass-loss rates. Due to dilatant processes caused by thermal expansion, which lead to the ‘hardening’ of the volume and closing of micro-cracks, UCS showed a continuous increment with temperatures up to 400 °C. On the other hand, the P-wave velocity and elastic modulus drop due to the opening of pre-existing cracks or the formation and expansion of new cracks as the temperature rises [64].

Decay zone (400–800 °C): is distinguished by complex factors that deteriorate the granodiorite properties. The granodiorite is a crystalline rock composed of minerals like quartz, feldspar, biotite, etc. These minerals have various thermal expansion coefficients. Hence, their expansion, content, particle size, and degree of adhesion cause complicated physical and mechanical changes. Structural and crystal water evaporated, and thermal stress between granules caused minerals like quartz to expand, leading to the growth of existing micro-cracks in the same temperature range. As the temperature rises, rock cracking is induced by thermal stress, and an increase in interior pores is generated by water loss. In addition, chemical reactions happen when granodiorite is exposed to high temperatures (over 400 °C), causing severe alterations to microcracks and microstructures. For example, at temperatures exceeding 400 °C, biotite interacts with oxygen [65], altering the microstructure, and the color of granodiorite shifts to a shiny gray with a reddish hue (Figure 4). 

Additionally, the quartz transition (α-quartz to β-quartz) induces a 0.45% linear expansion of the quartz at around 575 °C, as shown by Ohno et al. [66], as confirmed by DTA analysis (Figure 6). That contributes to quartz grain volumetric expansion [67], resulting in structural alterations when thermally treating the granodiorite. As a result, these types of mineral alterations can cause microcrack formation and propagation, and as the temperature rises, the density and width of the fractures inside the granodiorite increase. Hence, the P-wave velocity values drop dramatically between 400 and 600 °C, and these findings are consistent with the porosity iterations as a function of temperature. 

On the other hand, thermal stress and new intergranular and intragranular microcrack formation due to high temperatures are highly noticeable in this zone (Figure 7c and Figure 9c). Thus, due to changes in the microstructure and mineral composition of granodiorite caused by thermal treatment, its macroscopic mechanical properties deteriorate. Consequently, UCS and E showed a severe decrease between 400 and 600 °C, and the thermal damage factor values for Vp and E hit their peak (Figure 14). A fracture network was created between (600–800 °C), and intergranular and transgranular microcracks were connected and fused (Figure 9d and Figure 10d). The higher the temperature a sample is exposed to, the more intense the mineral expansion occurs, and thermally enlarged minerals compress. Thus, the thermal expansion of various minerals within granodiorite, including quartz, plagioclase, K-feldspar, and biotite, is caused by these high temperatures. In addition, structural damage caused by these expansions causes irreversible distortion and induces structural damage. Additionally, in both experimental and TGA examinations of the granodiorite specimen, the maximum weight losses were observed in this temperature phase. Hence, that range of thermal treatments substantially influences granodiorite’s physical and mechanical characteristics and related microstructure. Thus, measurements of UCS were small, and Vp and E were impossible to measure.

## 5. Conclusions

The effects of temperature on Egyptian granodiorite’s microstructure deterioration were comprehensively investigated and analyzed using X-ray diffraction, X-ray fluorescence, thermal gravimetric analysis, differential thermal analysis, scanning electron microscopy, and optical microscopy techniques. The physical and mechanical properties responses were then linked to these analyses to determine how granodiorite mineralogy and microstructure evolved with temperature. From the outcomes of various physical, thermal, and mechanical properties, it can be supposed that the Egyptian granodiorite lies in three zones distinguished by different temperature ranges. The strengthening stage (up to 200 °C), the transition phase (200–400 °C), and the decay area (400–800 °C). Following are some key findings that may be drawn from these extensive measurements and our subsequent discussion:

(1) Microscopy analyses revealed intergranular microcracks at 200 °C and some transgranular microcracks at 400 °C ascribed to attached-water evaporation and thermal stress divergences between minerals. Hence, moderate deterioration in porosity, mass loss, and P-wave velocities were observed at this temperature range because the water content of granodiorite was limited, as approved by TGA. On the other hand, trans-crystalline cracks rose by the thermal deformation of crystal grains at temperatures above 400 °C. As a result, a sharp reduction of granodiorite physical properties after the transition temperature (400 °C).
(2) Beyond 400 °C, the thermochemical processes accelerated, and structural and crystal water evaporated, increasing the porosity exponentially. Hence, granodiorite characteristics deteriorate significantly, presumably due to various microcracks, the quartz α-β transition at 575 °C, according to the DTA examination. Furthermore, biotite reactions with oxygen-induced color change from lightening to pale grey with a reddish hue, and the quartz α-β transition at 575 °C, according to the DTA examination.
(3) The UCS of granodiorite samples climbed first due to thermal hardening up to 400 °C; in contrast, Young’s modulus decreased when the heat treatment temperature increased. Meanwhile, UCS and E declined exponentially between 400 °C and 600 °C because of crack expansion, rising differential thermal expansion of minerals, and dehydration. Consequently, at 800 °C, the UCS dramatically reduced due to the interactions and coalescences of the intergranular and transgranular fractures, explaining why E and Vp could not be measured.
(4) Three main failure modes of granodiorite can be linked to the initiation and propagation of thermal microcracks. At temperatures below 400 °C, intergranular microcracks were visible, while some transgranular microcracks appeared, and the axial splitting mode occurred. At the same time, the growth of existing micro-cracks at 600 °C led to shear mode. Forming a fracture network of intergranular and transgranular microcracks at 800 °C contributed to the complex failure mode.
(5) Egyptian granodiorite has a thermal transition zone from strengthening to decay zone between 200 and 400 °C, which means 400 °C is the temperature threshold. Following this temperature, P-wave velocity and rock mass decreased significantly, porosity doubled, and the evolution of transgranular cracks was revealed via SEM and OM, resulting in a substantial decline in the UCS and E.

This investigation provides an in-depth insight into the modifications induced by temperature on Egyptian granodiorite microstructure and the affiliated mineralogical, physical, and mechanical responses. As a result, the temperature phases mentioned in this study must be considered when using granodiorite in thermal applications, preventing structural collapse and huge socioeconomic harm.

## Figures and Tables

**Table 1:** XRF analysis of the main oxide components of granodiorite at target temperatures.

Oxide (%)/Temp (°C)	RT	200	400	600	800
SiO_2_	61.60	61.20	59.76	60.48	61.20
Al_2_O_3_	16.80	16.60	15.90	16.30	16.70
Fe_2_O_3_	6.50	7.54	9.94	8.88	6.32
CaO	6.71	6.61	6.44	6.72	6.97
Na_2_O	3.09	2.96	2.99	3.17	3.11
MgO	2.37	2.31	2.18	2.04	2.62
K_2_O	1.28	1.15	1.13	1.39	1.39

## Data Availability

Data are contained within the article.
